# Detection of cerebral aneurysms using artificial intelligence: a systematic review and meta-analysis

**DOI:** 10.1136/jnis-2022-019456

**Published:** 2022-11-14

**Authors:** Munaib Din, Siddharth Agarwal, Mariusz Grzeda, David A Wood, Marc Modat, Thomas C Booth

**Affiliations:** 1 School of Biomedical Engineering & Imaging Sciences, King's College London, London, UK; 2 Department of Neuroradiology, King's College Hospital NHS Foundation Trust, London, UK

**Keywords:** Aneurysm, Angiography, Brain, CT Angiography, Magnetic Resonance Angiography, artificial intelligence, deep learning, machine learning

## Abstract

**Background:**

Subarachnoid hemorrhage from cerebral aneurysm rupture is a major cause of morbidity and mortality. Early aneurysm identification, aided by automated systems, may improve patient outcomes. Therefore, a systematic review and meta-analysis of the diagnostic accuracy of artificial intelligence (AI) algorithms in detecting cerebral aneurysms using CT, MRI or DSA was performed.

**Methods:**

MEDLINE, Embase, Cochrane Library and Web of Science were searched until August 2021. Eligibility criteria included studies using fully automated algorithms to detect cerebral aneurysms using MRI, CT or DSA. Following Preferred Reporting Items for Systematic Reviews and Meta-Analysis: Diagnostic Test Accuracy (PRISMA-DTA), articles were assessed using Quality Assessment of Diagnostic Accuracy Studies 2 (QUADAS-2). Meta-analysis included a bivariate random-effect model to determine pooled sensitivity, specificity, and area under the receiver operator characteristic curve (ROC-AUC). PROSPERO: CRD42021278454.

**Results:**

43 studies were included, and 41/43 (95%) were retrospective. 34/43 (79%) used AI as a standalone tool, while 9/43 (21%) used AI assisting a reader. 23/43 (53%) used deep learning. Most studies had high bias risk and applicability concerns, limiting conclusions. Six studies in the standalone AI meta-analysis gave (pooled) 91.2% (95% CI 82.2% to 95.8%) sensitivity; 16.5% (95% CI 9.4% to 27.1%) false-positive rate (1-specificity); 0.936 ROC-AUC. Five reader-assistive AI studies gave (pooled) 90.3% (95% CI 88.0% – 92.2%) sensitivity; 7.9% (95% CI 3.5% to 16.8%) false-positive rate; 0.910 ROC-AUC.

**Conclusion:**

AI has the potential to support clinicians in detecting cerebral aneurysms. Interpretation is limited due to high risk of bias and poor generalizability. Multicenter, prospective studies are required to assess AI in clinical practice.

**WHAT IS ALREADY KNOWN ON THIS TOPIC**

- Aneurysm detection using artificial intelligence (AI) has been described as a primary focus in the field of neurointervention, but there has been no comprehensive systematic review or meta-analysis of relevant studies to assess their suitability for clinical use.

**WHAT THIS STUDY ADDS**

- Most studies had a high risk of bias with poor generalizability (only 11/43 studies (26%) used ideal reference standards, and 6/43 studies (14%) used external test sets). AI tools for aneurysm detection are not ready for incorporation into routine clinical practice because of these reasons as well as the low level of evidence supporting their use, and AI performance being compromised by high false-positive rates: while univariate *per-aneurysm analysis* of 22 studies gave an 89.0% pooled true-positive rate, the high false-positive rate means that each examination will produce several aneurysm candidates requiring review, plausibly leading to an increase in workload and cost.
- Nonetheless, their eventual use in the clinic is possible given that bivariate *per-patient analysis* of six studies using standalone AI, and five studies using reader-assistive AI, gave 0.936 and 0.910 area under the receiver operating characteristic curve, respectively.

**HOW THIS STUDY MIGHT AFFECT RESEARCH PRACTICE OR POLICY**

- To ensure clinical adoption, large and representative datasets should be used in studies developing AI tools, with subsequent clinical validation achieved through prospective multicenter studies.

## Introduction

Cerebral aneurysm rupture is the most common cause of non-traumatic subarachnoid hemorrhage, accounting for 85% of cases.^1^ Aneurysmal subarachnoid hemorrhage (aSAH) yields a poor prognosis, with a mortality rate of up to 44%.^2^ There is also a large morbidity burden with up to a fifth of surviving patients becoming functionally dependent.^2^

Aneurysms are common with an estimated prevalence of 3.2% in the general population, but may be higher among females, the elderly, those with a strong family history of aneurysm formation, certain genetic conditions, smokers and those with hypertension.^3^ The early identification of aneurysms provides the opportunity for expert rupture risk stratification to allow the optimal course of management to be expedited with the aim of improving outcomes.^4^ If optimal management requires treatment, this may be endovascular embolization or surgical clipping.

There are two common indications where the accurate detection of cerebral aneurysms is required. One is following aSAH, where the ruptured aneurysm needs to be detected. Another is when an unruptured aneurysm is an incidental finding—for example, during vascular imaging following a stroke or transient ischemic attack. Screening may also occur in high-risk populations.

The reference standard imaging modality to detect cerebral aneurysms is digital subtraction angiography (DSA). However, computed tomography angiography (CTA) and magnetic resonance angiography (MRA) are regularly used in clinical practice due to their less invasive nature.^5^ As the global volume of scans performed increases annually, it is becoming increasingly challenging for the radiology community to meet the reporting demand, impacting human factors such as fatigue.^6 7^ Many cerebral aneurysms can be challenging to discern, and many can be time-consuming to identify. Together, these factors can contribute to diagnostic errors. Artificial intelligence (AI) computer-assisted diagnosis (CAD) tools may help tackle these challenges as they have shown promise as diagnostic biomarkers in accurately and efficiently detecting aneurysms using machine learning.^8^ In clinical practice, such decision support software can be standalone (in place of a reader) or be used to assist the reader. While numerous AI CAD tools have been developed, it is currently unclear how well these perform in clinical practice. The aim of this study is to systematically review and perform a meta-analysis of the diagnostic accuracy of AI CAD diagnostic biomarkers in detecting cerebral aneurysms. This will highlight the current developments in the field, help to direct future research and ultimately guide clinical practice.

## Materials and methods

This systematic review and meta-analysis are PROSPERO (International prospective register of systematic reviews) registered (CRD42021278454). The review followed Preferred Reporting Items for Systematic Reviews and Meta-Analysis: Diagnostic Test Accuracy (PRISMA-DTA),^9^ informed by Cochrane review methodology regarding developing study inclusion criteria,^10^ study search,^11^ and quality assessment.^12^

### Search strategy and selection criteria

A sensitive search with low precision was undertaken comprising subject headings with exploded terms, without language restrictions.^11^ Search terms were applied to Embase, MEDLINE, Web of Science, and the Cochrane Register to extract original research articles published until August 2021 (online supplemental table S1). The bibliography of all relevant articles was screened to capture additional articles. Pre-prints and non-peer reviewed articles were excluded.

10.1136/jnis-2022-019456.supp1 Supplementary data

#### Inclusion criteria

Included studies consisted of primary research studies, employing brain MRA, CTA or DSA datasets, applying automated AI algorithms, and detecting cerebral aneurysms as the target condition.

#### Exclusion criteria

Excluded studies were those that used other imaging modalities, used no automated algorithm (in the extraction or selection of features, or in classification/regression), without an English language translation,^13 14^ or animal studies.

#### Index test and reference standard

The index test was the automated AI model detecting cerebral aneurysms. The reference standard was angiography (DSA, CTA or MRA) and the interpretation (report or image re-review; sole or consensus reading). Two individuals (MD and SA, radiologist-clinician, 1 and 4 years neuroimaging research experience, respectively) independently performed the literature search and selection.

### Data extraction and risk of bias assessment

Study quality, focusing on the risk of bias and concerns regarding applicability, was assessed using Quality Assessment of Diagnostic Accuracy Studies 2 (QUADAS-2) methodology^15^ tailored to the review question, incorporating items from the Checklist for Artificial Intelligence in Medical Imaging (CLAIM).^16^

Data extracted included: patient demographics; eligibility criteria; dataset imaging modality; scanner manufacturer and model; index test AI algorithm; reference standard employed; and information on training and test sets. Test sets were classified as either ‘internal’ or ‘external’. External test sets were acquired from a (geographically) different institution from where the training data were acquired. Internal test sets were acquired from the same institution. Details relating to hold-out and/or cross-validation methodology, as well as temporal splits where the training and test data were collected from separate periods, were captured. Data were also grouped according to whether the decision support software was tested in standalone or reader-assistive mode.

### Data synthesis and statistical analysis

Primary outcome measures were AI diagnostic test accuracy metrics. Two units of analysis were used due to the nature of the data: ‘per patient’ and ‘per lesion (aneurysm)’. Based on the published study data, 2×2 confusion matrices were made for hold-out test sets from which the primary diagnostic accuracy measures of sensitivity (recall) and specificity were calculated. Where performance measures for both internal and external tests were available, the external test data were used to determine performance accuracy. Specificity was only derivable from studies that provided per-patient data as the number of true negatives is arbitrary on a per-lesion basis. Therefore, for meta-analysis, the studies were divided into two groups: the first group (A) involved studies with sensitivity and specificity per-patient values; and the second group (B) involved studies with only sensitivity per-lesion values. The area under the receiver operating characteristics curve (ROC-AUC) values and the number of false-positive lesions per patient were extracted where available.

Secondary outcome measures were diagnostic test accuracy metrics of radiologists using AI; therefore ‘reader’ and ‘reader & AI’ performance metrics were also obtained. The term ‘reader’ was applied to any appropriately trained individual interpreting the imaging.

Data were extracted and quality assessment was performed independently by two reviewers (MD and SA). Disagreements were resolved through discussion, with any final arbitration through a third reader (TCB, neuroradiologist, 13 years AI research experience).

#### Meta-analysis

For group A, the meta-analysis’s principal diagnostic accuracy measures were sensitivity and specificity. A bivariate random-effect model^17^ (online supplemental material) was used to determine two pooled primary measures of accuracy: the true-positive rate (sensitivity/recall), and the specificity. Parameters of the bivariate random-effect model also allowed for the estimation of the summary ROC (SROC) curve and the SROC-AUC. Using a resampling approach^18^ model, estimates were used to obtain the pooled measures of balanced accuracy, the positive and negative likelihood ratios, and the diagnostic odds ratio.

Studies in group B underwent a univariate meta-analysis because they only contained data for sensitivity metrics. As all outcome measures included in this group were originally expressed as proportions of true-positives (sensitivities), the key results of meta-analysis (summary effect sizes) were also reported as pooled proportions.

Both group analyses used a linear random-effect model taking into account the possible true heterogeneity of effects across studies.^19^ The meta-analysis was conducted by a statistician (MG, 15 years of relevant experience). All the statistical analyses were performed in R (v 3.6.1). The R Package Mada (v 0.5.10)^20^ was used for the bivariate model.

## Results

### Characteristics of included studies and bias assessment

**Figure 1:**
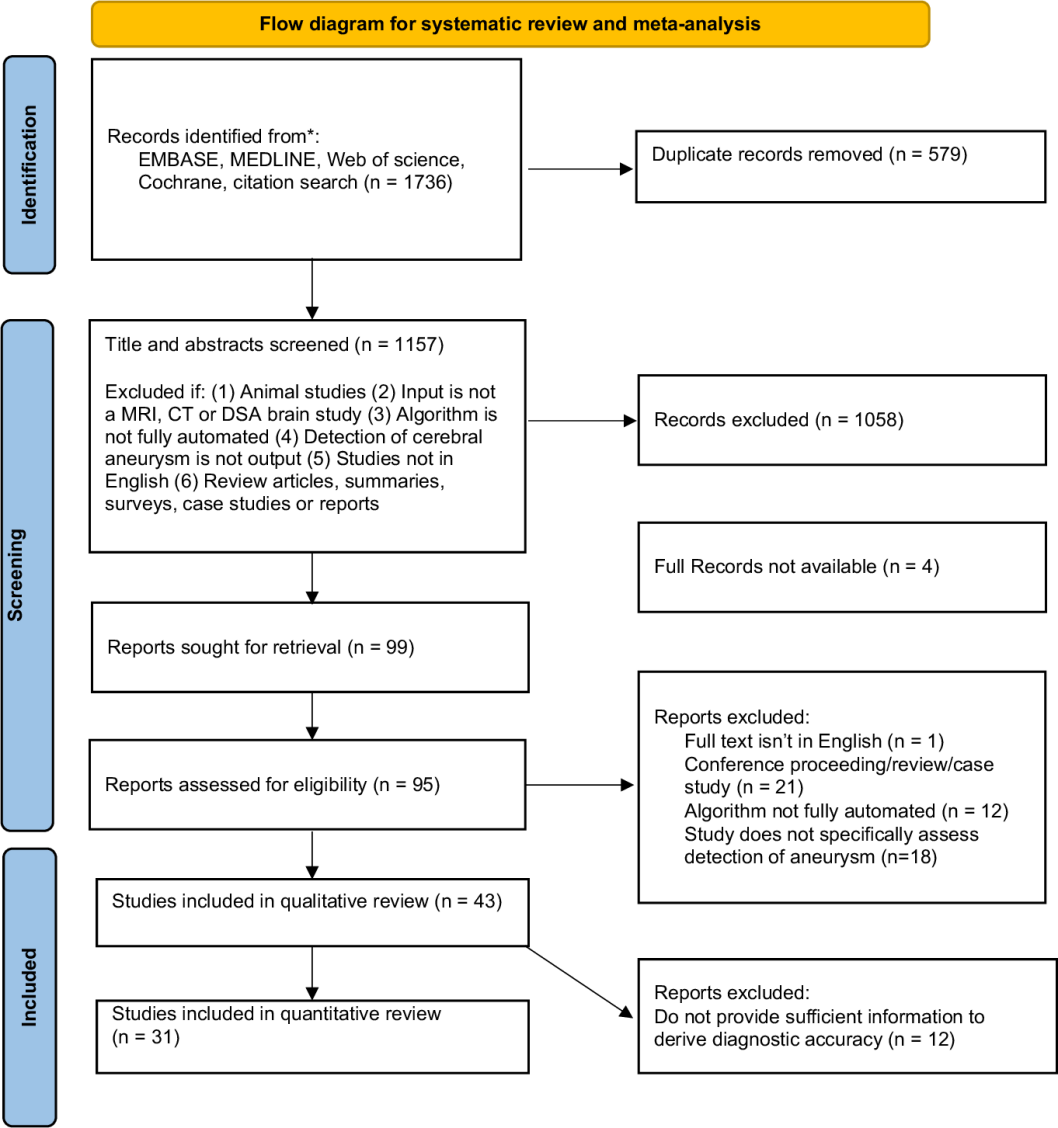
Flow diagram for systematic review and meta-analysis of cerebral aneurysm detection using artificial intelligence.

Figure 1 shows that overall, 1736 studies met the search criteria and 99 potentially eligible full-text articles were assessed. Forty-three studies ranging from October 2004 to August 2021 were included.^21–63^ The total number of patient cases used for both training and testing was 18 143, and of these 10 625 patients had aneurysms, with a combined total of 12 990 aneurysms (datasets that were used across different studies were only included once). Tables 1 and 2 detail the study characteristics and are presented as subgroups containing 34/43 (79%) ‘AI standalone’ and 9/43 (21%) ‘AI & reader’ studies, respectively. One ‘AI standalone’ study (1/34, 3%) was prospective. One ‘AI & reader’ study (1/9, 11%) was prospective and used AI CAD during clinical practice. The remaining studies (41/43, 95%) in both subgroups were conducted retrospectively, in a laboratory environment, thus providing limited evidence on its clinical validity.^64^ Eleven studies employed DSA (11/43, 26%), 6/43 (14%) CTA, 24/43 (56%) MRA, and 2/43 (5%) multi-modality datasets. There were 13/43 (30%) multicenter studies (dataset from two or more different sites). Twenty-six studies (26/43, 60%) used more than one scanner model, and 13/43 (30%) studies used scanners from more than one manufacturer. Nineteen studies (19/43, 44%) included only datasets where patients had aneurysms.

**Table 1:** Studies applying artificial intelligence as a standalone method for the automatic detection of cerebral aneurysms

Author	Modality	Study design	Demographics: total cases; aneurysm-positive cases (+); total aneurysms (A). If data available: % female; mean age; mean aneurysm diameter (range); rupture status	Reference standard	Index test	Dataset: number of cases (number of which contain aneurysms (+), if different from the total number of cases)	Type of test set	Test set performance(If available): lesion sensitivity; FP/case; PPV; NPV; accuracy; patient sensitivity; patient specificity; precision; AUC; F1 score
Nomura *et al* 2014^21^	MRA-TOF	Retrospective single center	2269 cases; 472+; 578A; 3 mm	2 radiologists reviewed MRA-TOF	cCAD: Gaussian filter with boosting algorithm (AdaC2)	Training=490 (362+); Test=1779 (110+)	Internal; temporal split	Lesion sensitivity: 95.2%; FP/case: 9
Jin *et al* 2016^22^	MRA-TOF	Retrospective single center	30 cases; 30+; 31A; 69.2 years; 67% female; 3.7 mm (2.0–5.5 mm); unruptured	2 neuroradiologists reviewed CTA/DSA	cCAD: ellipsoid convex enhancement filter	Test=30	Internal; LOOCV	Lesion sensitivity: 100%; FP/case: 31.8
Arimura *et al* 2006^23^	MRA-TOF	Retrospective multicenter	178 cases; 87+; 214A; 5.2 mm (1–15 mm); unruptured	2 neuroradiologists reviewed MRA-TOF and other available imaging	cCAD: 3D selective enhancement filter using Hessian matrix, shape-based difference image technique	Training=115 (53+); Training/test=63 (34+)	Internal; LOOCV	Lesion sensitivity: 94%;, FP/case: 2.3
Arimura *et al* 2004^24^	MRA-TOF	Retrospective single center	60 cases; 29+; 36A; 6.6 mm (3–26 mm); unruptured	NR	cCAD: 3D selective enhancement filter using Hessian matrix	Test=60 (29+)	Internal; LOOCV	Lesion sensitivity: 100%; FP/case: 2.4
Joo *et al* 2020^25^	MRA-TOF	Retrospective multicenter	744 cases; 644+; 761A; 3.2 mm; unruptured	2 neuroradiologists reviewed MRA-TOF	DL: 3D ResNet	Training and validation=468; Internal test=170 (120+). External test106 (56+)	External; geographical split	Lesion sensitivity: 85.7%; PPV: 91.5%; Accuracy: 88.5%; Patient specificity: 98.0%
Yang *et al* 2011^26^	MRA-TOF	Retrospective single center	287 cases; 92+cases; 147A; (1–31 mm); unruptured	1 general and 1 neuroradiologist reviewed DSA	cCAD: Dot enhancement filter	Test=92 (92+)	No training required	Lesion sensitivity: 96%; FP/case: 11.6
Timmins *et al* 2021^27^	MRA-TOF	Retrospective single center	300 cases; 254+; 282A; 75% female; 55 years; 3.6 mm (1.0–15.9 mm); unruptured	1 neuroradiologist and 1 trained reader reviewed MRA-TOF	DL: 3D CNN Retina U-net by MiBaumgartner	Training=113 cases (93+); Test=141 (115+)	Internal; hold out	Lesion sensitivity: 67%; FP/case: 0.13
Nakao *et al* 2018^28^	MRA-TOF	Retrospective single center	450 cases; 450+; 508A; 45% female; 61 years; 3 mm; unruptured	2 radiologists reviewed MRA-TOF	DL: Voxel based CNN (chainer 1.6.1)	Training=300; Validation=50; Test=100	Internal; temporal split	Lesion sensitivity: 94.2%; FP/case: 2.9
Hanaoka *et al* 2019^29^	MRA-TOF	Retrospective single center	300 cases; 300+; 300A	2 radiologists reviewed MRA-TOF	cCAD: HoTPiG (voxel-based feature set) and Hessian based features with single SVM	Training=200; Test=100	Internal; 3-fold CV	Lesion sensitivity: 80%; FP/case: 3
Faron *et al* 2020^30^	MRA-TOF	Retrospective multicenter	85 cases; 85+; 115A; 68% female; 56 years; 7.1 mm (2.1–37 mm); unruptured	1 neuroradiologist reviewed MRA-TOF and other available imaging	DL: DeepMedic CNN	Training=58; Validation=10; Test=17	Internal; CV (5-fold)	Lesion sensitivity: 90%; FP/case: 6.1
Sichtermann *et al* 2019^31^	MRA-TOF	Retrospective multicenter	85 cases; 85+; 115A; 68% female; 56 years; 7.1 mm (2.1–37 mm); unruptured	2 radiologists reviewed MRA-TOF and other available imaging	DL: DeepMedic CNN	Training=58; Validation=10; Test=17	Internal; CV (5-fold)	Lesion sensitivity: 90%; FP/case: 6.1
Hou *et al* 2020^32^	MRA-TOF	Retrospective multicenter	350 cases; 179+; 179A, 7.5 mm (2.4–23 mm); unruptured	NR	DL: 1D CNN by generating 1D vectors from MIP images	Training=245 (126+); Validation=35 (17+); Test=70 (36+)	Internal; hold out	Lesion sensitivity: 93.2%; Precision: 96.9%; Accuracy: 95.2%; AUC: 0.99;F1: 0.950
Allenby *et al* 2021^33^	MRA-TOF	Retrospective multicenter	623 cases; 21+; 21A; unruptured	1 interventional radiologist reviewed MRA-TOF	cCAD: single-voxel morphometry	Test=623 (21+)	No training required	Patient specificity: 86%; Sensitivity: 81%; FP/case: 0.14
Stember *et al* 2019^34^	MRA-TOF	Retrospective single center	336 cases; 336+; 336A; (3–23 mm)	Original radiological reports	DL: U-net CNN	Training=250; Test=86	Internal; hold out	Lesion sensitivity: 98.8%; AUC:0.87
Chen *et al* 2020^35^	MRA-TOF	Retrospective single center	131 cases; 131+; 140A, 63% female; 57 years; 6.5 mm; unruptured	2 radiologists reviewed DSA	DL: 3D-U-net CNN	Training=76; Validation=20; Test=35	Internal; hold out	Lesion sensitivity: 82.9%; FP/case: 0.86
Ueda *et al* 2019^36^	MRA-TOF	Retrospective multicenter	1271 cases; 1271+; 1477A; 72% female; 68 years; 4.1 mm	2 radiologists reviewed MRA-TOF	DL: ResNet-18 using skip connections	Training and validation=683; Internal test=521; External test=67	External; geographical split	Lesion sensitivity: 93%; FP/case: 5
Nomura *et al* 2021^37^	MRA-TOF	Retrospective multicenter	519 cases; 399+; 448A; 46% female; 3.1 mm	2 radiologists reviewed MRA-TOF	DL/cCAD: 3 software: (i) 3D local intensity structure analysis; (ii) graph-based; (iii) CNN	Training=399 (339+); Test set 1=60 (30+); Test set 2=60 (30+)	External; geographical split	Per patient or per lesion performance measurements not available
Nemoto *et al* 2017^38^	MRA-TOF	Retrospective single center	300 cases; 300+; 300A; 50% female; 59.8 years; 3.1 mm	2 radiologists reviewed MRA-TOF	cCAD: voxel and candidate classifier ensembles with cost-sensitive AdaBoost	Training=200; Test=100	Internal; 3-fold CV	Lesion sensitivity: 56.8%; FP/case: 10
Hainc *et al* 2020^39^	2D DSA	Retrospective single center	240 cases; 136+; 186A; 65% female, 59 years, 7 mm; ruptured and unruptured	2 neuro-interventional radiologists reviewed DSA	DL: commercial software by Cognex, ViDi Suite 2.0, Cognex Inc	Split via DSA Projections: 706 (335+) Training=565; Test=141	Internal; 45-fold CV	Lesion sensitivity: 79%; Specificity: 79%;F1: 0.77; Precision: 0.75; Mean AUC: 0.76
Zeng *et al* 2020^40^	2D DSA	Retrospective single center	300 cases; 250+; 263A	5 radiologists reviewed DSA	DL: 2D CNN with spatial information fusion (VGG16)	No test set	No separate test set	No test set data
Jin *et al* 2020^41^	2D DSA	Retrospective single center	493 cases; 493+; 1205A; 62% female; 55 years; 7.4 mm (1.3–40 mm)	2 neurologists reviewed DSA and third reader for arbitration	DL: end to end spatial temporal U-Net CNN (Keras-2.2.0 with TensorFlow-1.4.0 backend)	Training=249; Validation=98; Test=146	Internal; hold out	Lesion sensitivity: 89.3%; Patient sensitivity: 97.7%; FP/case: 3.77
Liu *et al* 2021^42^	3D DSA	Retrospective single center	451 cases; 451+; 485A; 61% female; 56 years; 7.1 mm	2 neuroradiologists reviewed DSA	DL: 3D-Dense-Unet CNN	Training=347; Validation=41; Test=63	Internal; hold out	Lesion sensitivity: 88.4%; FP/case: 0.61
Hu *et al* 2020^43^	3D DSA	Retrospective single center	145 cases; 145+; 165A; 66% female; 57.8 years	2 neuroradiologists reviewed DSA	cCAD: Bayesian optimized Hessian matrix filter	Test=145	No training required	Lesion sensitivity: 96.4%; Precision: 0.946; AUC: 0.98; F1 score: 0.955
Duan *et al* 2019^44^	2D DSA	Prospective single center	281 cases; 261+; 261A; 85% female; ruptured and unruptured	2 radiologists reviewed DSA	DL: CNN based on feature pyramid networks (using ResNet50)	Training=241; Test=40 (20+)	Internal; temporal split	Patient sensitivity: 96.0%, Specificity: 91.0%; Accuracy: 93.5%; AUC: 0.94; F1 score: 0.94
Rahmany *et al* 2018^45^	2D DSA	Retrospective single center	30 cases; 30+; 30A	2 neuroradiologists reviewed DSA	cCAD: Priori knowledge applied to fuzzy logic-based model with Fuzzy information fusion	Test=30	No training required	Results for 5 cases : Patient sensitivity: 100%; Specificity: 100%, Accuracy: 98.4%; AUC: 0.96
Rahmany *et al* 2019^46^	2D DSA	Retrospective single center	30 cases; 30+; 30A	2 neuroradiologists reviewed DSA	cCAD: LBP for feature extraction, and KNN classification	Training=20; Test=10	Internal; hold out	Per patient or per lesion performance metrics not available
Malik *et al* 2018^47^	DSA	Retrospective single center	59 cases; 47+; 47A; (6–21 mm); unruptured	1 radiologist reviewed DSA	DL: classification multi-layer perceptron neural network	Split into 210 ROI: Training=189; Test=21	Internal; 10-fold CV	Per patient or per lesion performance metrics not available
Chandra *et al* 2017^48^	2D DSA	Retrospective single center	15 cases; 15+; 15A	NR	cCAD: iterative double automated thresholding, morphological filtering	Test=15	No training required	Per patient or per lesion performance metrics not available
Khan *et al* 2019^49^	DSA	Retrospective single center	4 cases; 4+; 4A	NR	cCAD: sub-band morphological operation, gaussian filtering	Test=4	No training required	Per patient or per lesion performance metrics not available
Shi *et al* 2020^50^	CTA	Retrospective multicenter	1388 cases; 908+; 1145A; 31.3% female; 64 years; 4.4 mm; ruptured and unruptured	3 neuroradiologists reviewed DSA	DL: end-to-end 3D CNN (DAResUNet)	Training=927 (744+); Validation=100 (50+); Testing: internal=150 (75+); external=211 (39+)	External; geographical split	Lesion sensitivity: 76.1%; PPV: 49.3%; NPV: 95.8%; Accuracy: 81.0%; Patient sensitivity: 84.6%; Specificity: 80.2%; FP/case: 0.27
Shahzad *et al* 2020^51^	CTA	Retrospective single center	253 cases; 253+; 294A; 67% female; 55.1 years; ruptured and unruptured	1 neurosurgeon and 1 radiologist reviewed CTA and DSA (if available)	DL: 3D CNN based on DeepMedic	Training=68 (79+); Test=185 (215)	Internal; temporal split	Lesion sensitivity: 82%; F1: 0.66; Precision: 0.54; FP/case: 0.81
Dai *et al* 2020^52^	CTA	Retrospective multicenter	311 cases; 311+; 344A; 5.4 mm (1–24 mm)	1 radiologist reviewed CTA	DL: RCNN model and Resnet-50	Training=208; Test=103	Internal; hold out	Lesion sensitivity: 91.8%; FP/case 8.9
Hentschke *et al* 2014^53^	CE/TOF-MRA /CTA	Retrospective single center	151 cases; 81+; 112A; (2.0–5.5 mm); CTA: 72; CE-MRA: 38; TOF-MRA: 41; unruptured	2 neuroradiologists reviewed imaging	cCAD: sphere-enhancing filter and linear or non-linear classification	Test=151 (81+)	Internal; 4-fold CV	Lesion sensitivity: CE-MRA: 91%; TOF-MRA: 84%; CTA: 69%; FP/case: 10
Lauric *et al* 2010^54^	3D-RA/CTA	Retrospective single center	20 cases; 19+; 20A; (3.2–10 mm); 3D-RA: 10; CTA:10	2 readers reviewed DSA/CT	cCAD: 3D shape analysis using writhe number	Test=20 (19+)	No training required	Lesion sensitivity: DSA: 100%; CTA: 100%; FP/case: DSA: 0.66; CTA: 5.36

+, aneurysm-positive cases; A, aneurysm; AUC, area-under-curve; cCAD, conventional computer assisted diagnosis; CE, contrast enhanced; CNN, convolutional neural network; CTA, CT angiography; CV, cross-validation; 1D, one dimensional; 2D, two dimensional; 3D, three dimensional; DL, deep learning; 3D-RA, three dimensional rotational angiography; DSA, digital subtraction angiography; FP/case, false-positives per case; HoTPiG, histogram of triangular paths in graph; KNN, k-nearest neighbour; LBP, local binary patterns; LOO, leave-one-out; MIP, maximum intensity projection; ML, machine learning; MRA-TOF, MR angiography-time of flight; NPV, negative predictive value; NR, not recorded; PPV, positive predictive value; ROI, regions of interest; SVM, support vector machine.

**Table 2:** Studies applying artificial intelligence as a reader-aid for the detection of cerebral aneurysms

Author	Modality	Demographics: total cases; aneurysm-positive cases (+); total aneurysms (A). If data available: % female; mean age; mean aneurysm diameter (range); rupture status	Study design	Dataset: number of cases (number of which contain aneurysms (+), if different from the total number of cases)	Reference	Index test used	Type of test set	AI performance: if available: lesion sensitivity; FP/case; accuracy; patient sensitivity; patient specificity; AUC	Reader performance: If available: lesion sensitivity; FP/case; accuracy; patient sensitivity; patient specificity; AUC	Reader+AI performance: If available: lesion sensitivity; FP/case; accuracy; patient sensitivity; patient specificity; AUC	Position of AI in pipeline
Miki *et al* 2016^55^	MRA-TOF	2701 cases; 189+; 203A; 38% female; 54 years (median)	Prospective single center	Test=2701 (189+)	2 radiologists reviewed MRA, with CAD assistance	cCAD: Gaussian filter based on Nomura *et al* 2014^24^ (see table 1)	Internal; temporal split	Lesion sensitivity: 82%	Lesion sensitivity: 64%	Lesion sensitivity: 69%	2nd reader
Štepán-Buksakowska *et al* 2014^56^	MRA-TOF	48 cases; 9+; 11A; 3.1 mm	Retrospective multicenter	Test=48 (9+)	2 neuroradiologists reviewed MRA-TOF and DSA	cCAD: Dot enhancement filter based on Yang *et al* 2011^26^ (see table 1)	External; Geographical split	Lesion sensitivity: 91%	Patient sensitivity: 70.4%; specificity: 79.5%; accuracy: 77.8%; AUC: 0.66	Patient sensitivity: 83.4%; patient specificity: 75.7%; accuracy: 77.1%; AUC: 0.76	1st reader
Miki *et al* 2020^57^	MRA-TOF	250 cases; 100+; 104A; unruptured	Retrospective single center	Test=250 (100+)	2 radiologists reviewed MRA-TOF	DL: Voxel based CNN based on Nakao *et al* 2018^28^ (see table 1)	Internal; hold out	Lesion sensitivity: 92.3%	NR	Lesion sensitivity: 71%; lesion specificity: 92%; AUC 0.89	Unclear
Kakeda *et al* 2008^58^	MRA-TOF	50 cases; 16+; 16A; 54% female, 64 years; 6.6 mm (3–26 mm); unruptured	Retrospective multicenter	Test=50 (16+)	2 neuroradiologists reviewed MRA and other available imaging	cCAD: 3D selective enhancement filter using Hessian matrix based on Arimura *et al* 2006^23^ (see table 1)	Internal; LOOCV	Lesion sensitivity: 81% FP/case: 2.7	Patient sensitivity: 75.8%; patient specificity: 82.7%; AUC: 0.85	Patient sensitivity: 80.9%; patient specificity: 88.6%; AUC: 0.90	2nd reader
Hirai *et al* 2005^59^	MRA-TOF	50 cases; 22+; 68% female; 59 years; 7.1 mm (3–26 mm); unruptured	Retrospective single center	Test=50 (22+)	2 neuroradiologists reviewed MRA and other available imaging	cCAD: 3D selective enhancement filter using Hessian matrix based on Arimura *et al* 2004^24^ (see table 1)	Internal; temporal split	NR	AUC: 0.93	AUC: 0.98	2nd reader
Sohn *et al* 2021^60^	MRA-TOF	332 cases; 135+; 169A; 64% female, 62 years; 4 mm (2–17 mm); unruptured	Retrospective single center	Test=332 (135+)	3 neuroradiologists reviewed MRA other available imaging	DL: 3D ResNet based on Joo *et al* 2020^25^ (see table 1)	Internal; temporal split	Lesion sensitivity: 92.3%. FP/case: 0.12; patient sensitivity: 74.8%; specificity: 93.9%	Lesion sensitivity: 74.8%; patient sensitivity: 73.5%; patient specificity: 94.8%	Lesion sensitivity 95.0%; patient sensitivity: 86.5%; patient specificity 95.2%	2nd reader
Park *et al* 2019^61^	CTA	818 cases; 328+; 358A; 64% female; 58 years; unruptured	Retrospective single center	Training=611 (223+); Validation=92 (46+); Test=115 cases (56+)	1 neuroradiologist reviewed CTA and DSA (if available)	DL: 3D CNN HeadXNet	Internal; hold out	Patient sensitivity: 94.9%; patient specificity: 66.1%; accuracy: 80.9%	Patient sensitivity: 83.1%; specificity: 96.0%; accuracy: 89.3%	Patient sensitivity: 89.0%; patient specificity: 97.5%; accuracy: 93.2%	1st reader
Pennig *et al* 2021^62^	CTA	172 cases; 172+; 205A; 63% female; 55.4 years; ruptured and unruptured	Retrospective single center	Training and validation=68; Test=104	1 neurosurgeon and 1 radiologist reviewed CTA and DSA (if available)	DL: ensemble model based on Shahzad *et al* 2020^51^ (see table 1)	Internal; temporally distinct	Lesion sensitivity: 85.7%; FP/case: 0.84	Lesion sensitivity: 88.1%	Lesion sensitivity: 97.1%	2nd reader
Yang *et al* 2021^63^	CTA	1468 cases; 1256+; 1543A; 48% female; 57 years; 4.1 mm (1–22 mm); ruptured and unruptured	Retrospective multicenter	Training=534; Validation=534; Test=400 (188+)	2 radiologists reviewed CTA	DL: 3D CNN ResNet-18	Internal; temporally distinct	Lesion sensitivity: 97.5% FP/case: 13.8 (internal validation set)	Lesion sensitivity: 79.1%; patient sensitivity: 81.6%; specificity: 95.9%; AUC: 0.60	Lesion sensitivity: 88.9%; patient sensitivity: 91.9%; specificity: 90.9%; AUC: 0.61	Unclear

+, aneurysm-positive cases; A, aneurysm; AI, artificial intelligence; AUC, area-under-curve; CTA, CT angiography; CV, cross-validation; 2D, two dimensional; 3D, three dimensional; DL, deep learning; DSA, digital subtraction angiography; FP/case, false-positives per case; LOO, leave-one-out; MRA-TOF, MR angiography-time of flight; NR, not recorded.

#### Reference standards

In 13/43 (30%) studies, the reference standard imaging modality of DSA imaging was used. The remainder used either MRA, CTA or a combination of imaging modalities. At least two independent readers were employed to determine the reference standard in 29/43 (67%) studies. The remainder used one reader or solely based the reference standard on the initial radiological reports.

#### Algorithm

Approximately half the studies (23/43, 53%) used a deep learning (DL) methodology, particularly convolutional neural networks (CNNs). The remainder used CAD systems employing shape filters and/or classic machine learning techniques.

#### Test sets

Six studies (6/43, 14%) used an external test set. Nineteen studies (19/43, 44%) used an internal hold-out test set, of which 8/43 (19%) studies employed a temporal split. Cross-validation (CV) alone was performed in 10/43 (23%) studies. One study did not have a test set, nor did it undergo cross-validation. The remaining 7/43 (16%) studies did not use training data because they used mathematical models utilizing filters for their algorithm.

#### AI standalone (subgroup)

For those studies (25/34, 74%) which gave results using per-lesion analysis, lesion sensitivities ranged from 0.67 to 1.0. Where available, the number of false-positive lesions per scan ranged from 0.13 to 31.8. Results from 6/34 (18%) studies were not comparable as five did not provide performance metrics on a per-patient or per lesion basis, and one did not use a test set.

#### AI & reader (subgroup)

Per-patient sensitivity and specificity were reported in 5/9 (56%) studies using a mean of the performance of the participating radiologists, with and without AI CAD assistance. These studies showed that there was an improved sensitivity when the imaging was interpreted with AI CAD assistance.

The proposed position of the AI CAD in the clinical pipeline varied among the nine studies. Five of nine studies (56%) placed the AI CAD as a ‘second reader’ which means that the radiologist initially reviewed the imaging without assistance, and then used the AI CAD to highlight lesion candidates. Conversely, 2/9 (22%) studies placed the AI CAD as a ‘first reader’, which means that the AI CAD had already highlighted the suspected lesion candidates on the images before the radiologist interpretation. The remaining 2/9 (22%) studies were unclear regarding the position of the AI CAD in the clinical pipeline.

#### Bias assessment and concerns regarding applicability

An analysis of the risk of bias assessment and concerns regarding applicability was performed for each study and summarized (online supplemental figure 1). Notably, there was a high risk of bias relating to the index test in 74% (32/43) of studies, mainly due to validation design, and 65% (28/43) for the reference standard, largely due to reader strategy and modality used. Regarding concerns of study applicability, these were high or unclear in 95% (41/43) of studies for patient selection. Six studies (6/43, 14%) did not explicitly mention their inclusion or exclusion criteria, and 22/43 (51%) studies excluded patients based on factors that could increase selection bias, such as aneurysm size, type, location, or presence of comorbidities.

10.1136/jnis-2022-019456.supp2 Supplementary data

#### Temporal analysis

The studies were divided into two groups depending on the year of publication. The first group consists of 13 studies that were published before 2018 (pre-2018), and the second group consists of 30 studies that were published in 2018 or later (post-2018). This arbitrary cut-off was chosen as it mirrors the notable observation that in 2018, arXiv (a repository where computer science papers are self-archived before publication in a peer reviewed journal) surpassed 100 new machine learning pre-prints per day.^65 66^ In the pre-2018 group, the lesion sensitivities ranged from 56.8% to 100% with false-positives/case ranging from 2.3 to 31.8. Whereas in the post-2018 group, the lesion sensitivities range from 67% to 100%, with a false-positives/case ranging from 0.12 to 13.8. While there is an impression of a slightly lower false-positive rate in more recent studies, comparison is limited as both groups have a similar high degree of bias and concerns regarding applicability (online supplemental figures 2 and 3). For example, 77% of pre-2018 studies had a high or unclear risk of bias for the index test used, compared with 73% for post-2018 studies; and 69% of pre-2018 and 60% of post-2018 studies had a high or unclear risk of bias for patient selection.

### Results of meta-analysis

**Figure 2:**
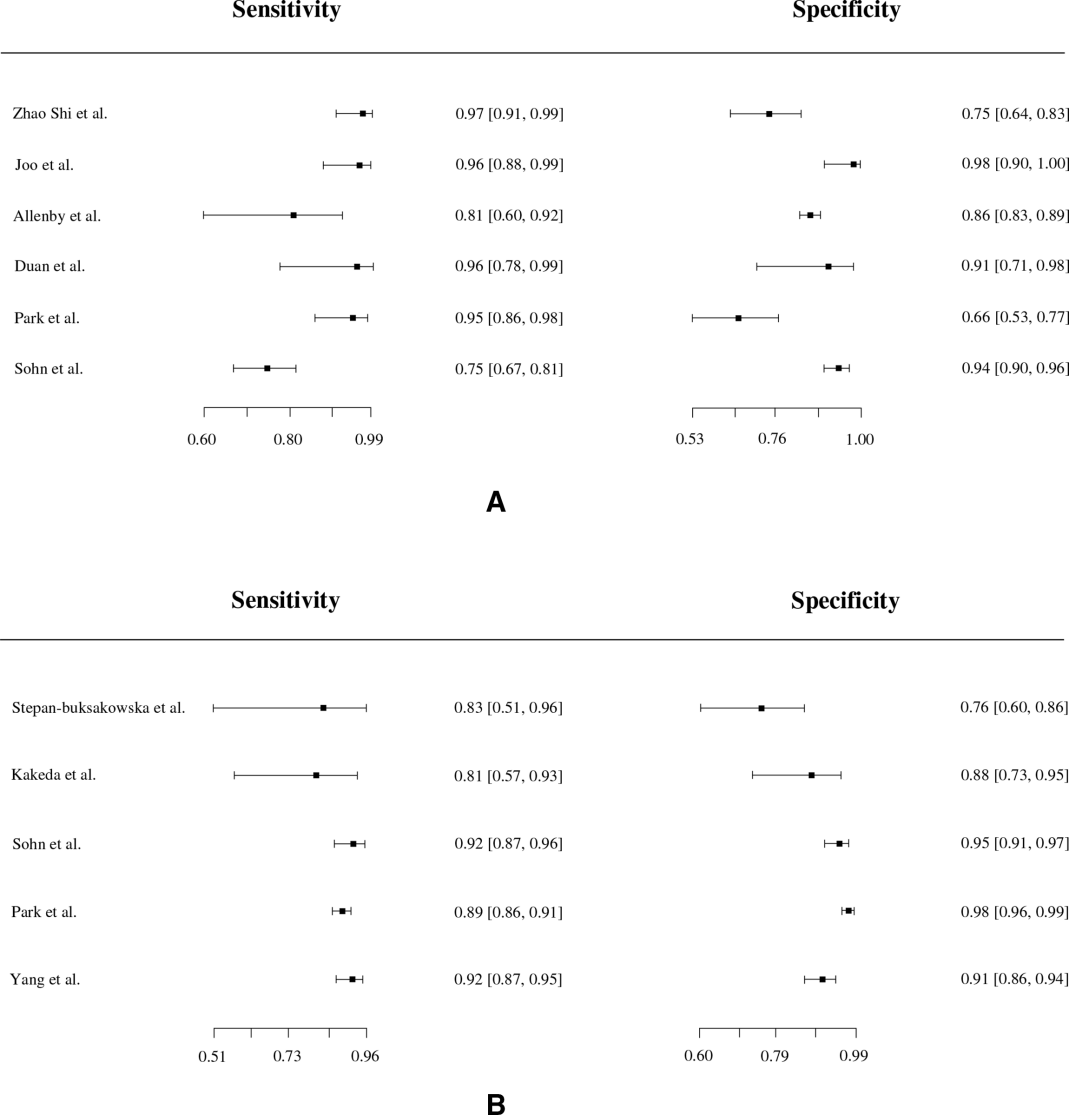
Forest plots of sensitivity and specificity of detection of cerebral aneurysms using artificial intelligence (AI). (A) AI standalone subgroup. (B) AI & reader subgroup.

**Figure 3:**
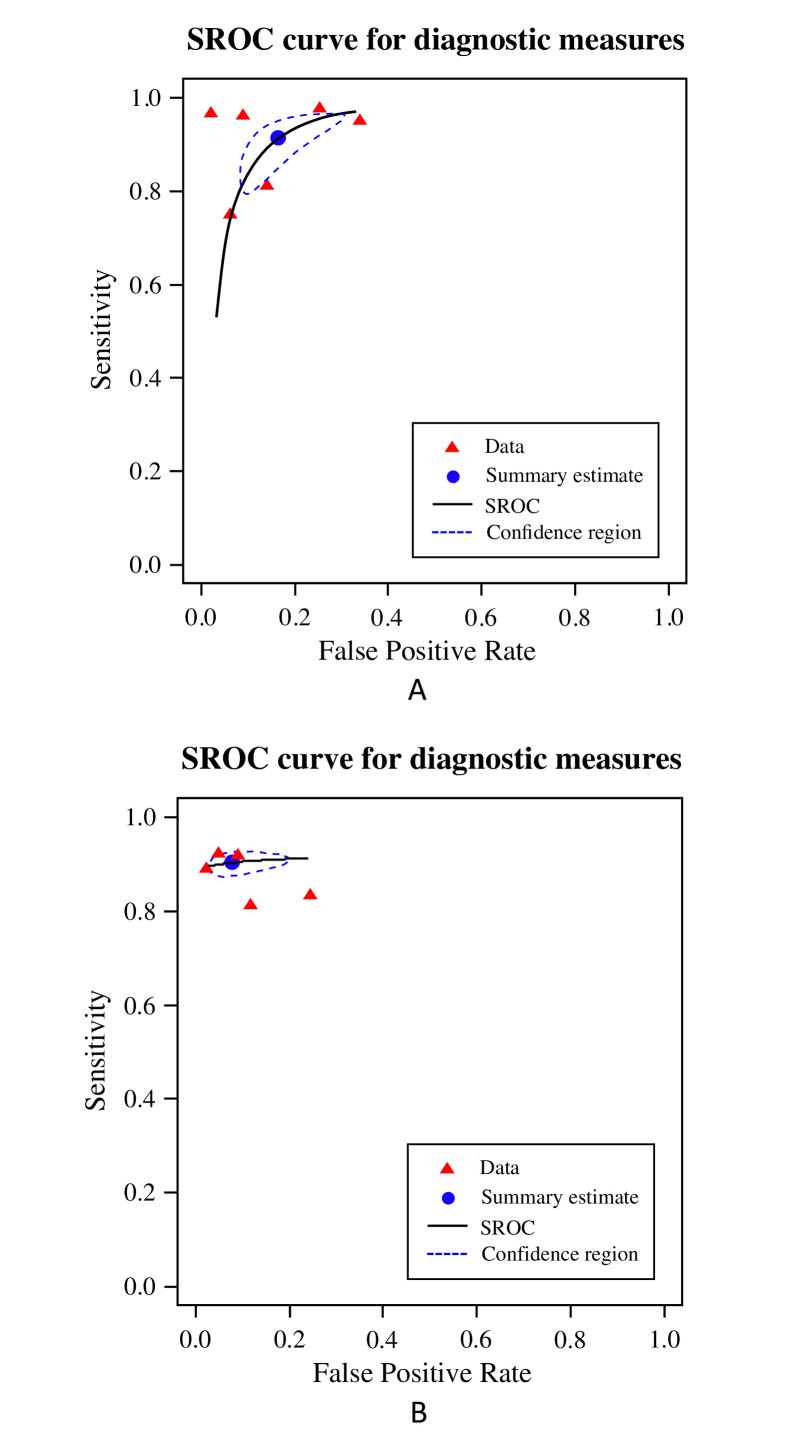
Summary receiver operator characteristic curve (SROC) for detection of cerebral aneurysms using artificial intelligence (AI). (A) AI stand-alone subgroup. (B) AI & reader subgroup. The summary point estimate and surrounding 95% confidence region is shown.

Group A consisted of 9/43 (21%) studies that were eligible for inclusion in a meta-analysis of per-patient diagnostic accuracy. These were divided into two subgroups: ‘AI standalone’ (6/9, 67% studies) and ‘AI & reader’ (5/9, 56% studies). Two studies (2/9, 22%) were included in both subgroups as they provided performance metrics for both categories. Forest plots of sensitivity and specificity (figure 2) graphically showed a high degree of heterogeneity. Additionally, χ^2^ tests were applied separately to both primary measures to statisically verify this hypothesis. For the ‘AI standalone’ subgroup, the P values were <0.001 for both sensitivities and specificities. For the ‘AI & reader’ subgroup, the P values were 0.43 and <0.001 respectively. Therefore, the bivariate random-effect model was ideal given the large level of heterogeneity between studies. For the ‘AI standalone’ subgroup, the pooled true-positive rate (sensitivity) was 91.2% (95% CI 82.2% to 95.8%) and the pooled false-positive rate (1-specificity) was 16.5% (95% CI 9.4% to 27.1%). For the ‘AI & reader’ subgroup, the pooled true-positive rate was 90.3% (95% CI 88.0% to 92.2%) and the pooled false-positive rate was 7.9% (95% CI 3.5% to 16.8%). A scatter plot of false-positive rates and true-positive rates (figure 3) demonstrates individual ROC point estimates and a summary ROC (SROC) giving an AUC of 0.936 and 0.910 for ‘AI standalone’ and ‘AI & reader’ studies, respectively.

**Figure 4:**
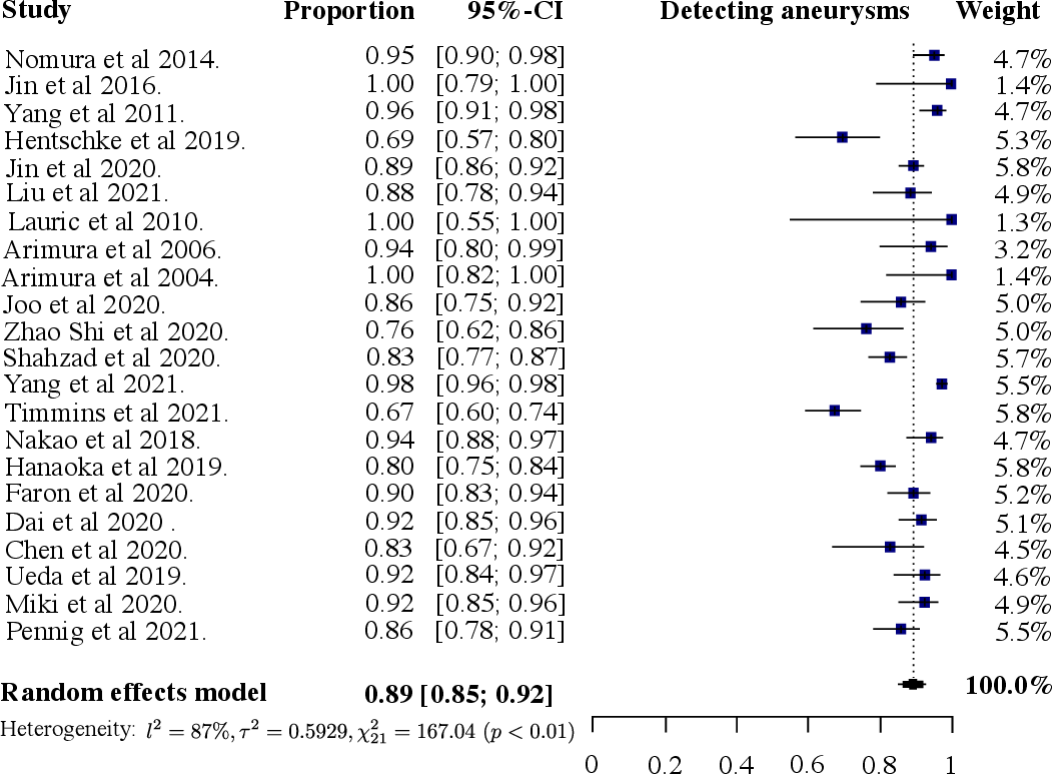
Forest plot of sensitivity for detecting intracranial aneurysms using a per-lesion analysis.

Group B included 22/43 (51%) studies that underwent univariate analysis of their per-lesion sensitivity metrics. The forest plot (figure 4) shows a high degree of heterogeneity, with an I^2^ of 87%. The χ^2^ test gave a P value <0.01. The pooled true-positive rate was 89% (95% CI 85% to 92%).

## Discussion

### Summary of findings

Current evidence for determining the diagnostic accuracy of AI in detecting cerebral aneurysms is of relatively low level.^67^ The validity of diagnostic accuracy is limited due to a high risk of bias and concerns regarding applicability across several domains; approximately half of studies had selective eligibility criteria that excluded patients based on aneurysm morphology or clinical characteristics, and few studies used an external test set while assessing the index test. Approximately half the studies employed DL methodology including CNNs as index tests.

### Limitations

#### Studies assessed

Most studies employed retrospective, single-center, and case–control designs using relatively small and enriched samples. Eligibility criteria varied among studies. Some excluded patients with aneurysms of a certain type, size, or other comorbidities. Several studies only included patients who had aneurysms, with no normal participants. These patient selection factors lead to spectrum bias^68^ and limit the generalizability of the results to a real clinical environment.

There were also limitations regarding index test evaluation. Most studies used internal hold-out test sets, often without temporal separation, or cross-validation to evaluate their model, as opposed to external test sets which provide a more accurate indication of how the model generalizes to other hospitals.^69 70^ Furthermore, it was unclear in most studies whether data leakage was prevented at the patient level from follow-up imaging.

Fewer than a third of studies used DSA as their reference standard, considered the ideal reference standard imaging modality for aneurysm detection.^5^ MRA and CTA were used as reference standards in the remainder of the studies, potentially leading to a systematic error with overestimation of model performance, given that it is plausible that not all aneurysms were identified. However, in routine clinical practice cross-sectional angiography is an acceptable first-line diagnostic biomarker because it is non-invasive with good accuracy. Therefore, an AI CAD index test benchmarked against a cross-sectional angiography reference standard demonstrating high-performance accuracy may have clinical applicability, provided that there is adherence to other aspects of reference standard methodology, including using at least two readers.

Another consideration is whether aneurysm rupture status impacts the diagnostic accuracy of AI CAD models including the interaction of aSAH on AI standalone and AI reader assist results. Unfortunately, not all studies mentioned rupture status (17/34 (50%) in AI standalone; 7/9 (78%) in AI reader assist), few contain ruptured aneurysms (4/34 (12%) in AI standalone; 2/9 (22%) in AI reader assist), and fewer still published detailed data precluding a meaningful analysis in this systematic review. For now, there are limited data. One study tested their AI model exclusively on ruptured cerebral aneurysms and found their accuracy to be comparable to other models which used only unruptured cerebral aneurysms.^51^ A follow-on study investigated the performance of the same model as a reader aid in aSAH cases and concluded the same.^62^ A third study also found no significant difference in their standalone model’s performance in cases with and without aSAH.^50^ Where possible, future studies should provide a comparison of their model’s performance on both ruptured and unruptured cerebral aneurysms. However, regardless of diagnostic accuracy, definitive DSA in almost any case of spontaneous subarachnoid hemorrhage is mandated, AI CAD or not.^71^

#### Review process

Reference standard heterogeneity was also introduced with variable levels of expertise among readers and different labelling methodologies, ranging from using the original radiological report alone to several dedicated radiologists re-reviewing the imaging.

The exclusion of pre-prints may exacerbate publication bias. More data science-oriented teams may be less inclined to publish in a peer reviewed journal compared with more clinically-oriented teams, due to the mismatch between the speed of data science development and the peer review process.^66^

Our review includes studies which range across a large time period. AI methodology is changing at a rapid pace, and it could be argued that the older studies may not accurately reflect the diagnostic performance or quality of the more recent studies. Therefore, this could be a contributing factor to the heterogeneity in the analysis. However, on comparing the newer (post-2018) studies with older (pre-2018) studies, there is no demonstrable improvement in the study quality, with high levels of bias being present in both groups. Even though some recent studies may demonstrate better performance accuracy, the studies themselves are of insufficient quality. This emphasizes the importance for researchers to ensure that they are designing robust, high-quality studies when developing and validating their AI tools.

### Role in clinical pipeline

Based on current evidence, AI CAD are unlikely to be used as standalone readers and are more likely to assist radiologists during diagnosis. One key reason is their high false-positive rate, meaning that each examination will produce several aneurysm candidates requiring review, plausibly leading to an increase in workload and cost. This may make AI CAD systems less appealing to clinicians and healthcare systems.

Studies investigating the impact of AI CAD assisting radiologists implemented the tool in different parts of the clinical pipeline, but numbers were small precluding meaningful analysis. Studies directly investigating pipeline positioning are warranted, incorporating phenomena related to over-reliance of automation^72^ and error associated with ‘satisfaction of search’.^73–75^ Incremental benefit may be marked when radiologists are interpreting vascular imaging for indications typically unrelated to aneurysms (e.g., ischemic stroke) in centers without neuroradiologists.

### Current evidence in the field

Aneurysm detection has been described as a primary focus in the field of cerebral aneurysms and radiology.^76^ Our systematic review and meta-analysis provide evidence for the quality and performance accuracy of all published studies using AI CAD for aneurysm detection. Another recent systematic review and meta-analysis included 20 CNN studies to identify cerebral aneurysms.^77^ Our systematic review emphasizes the current low level of evidence which undermines the performance accuracy of reported studies including those using CNNs, whereas the review by Abdollahifard *et al* does not raise any concerns regarding the bias or applicability of the studies. The discrepancy is potentially because we systematically applied PRISMA-DTA and QUADAS-2 methodology, which is the standard used for diagnostic accuracy studies.^15^ We pooled primary measures of accuracy using bivariate random-effects methodology which accounts for the negative correlation of sensitivity and specificity and differing cut-off values between studies, and second, it accounts for a relatively high degree of heterogeneity in the results of diagnostic studies (online supplemental material). Despite the authors not performing such an analysis, they conclude that CNN models would be best placed to assist readers rather than acting independently, due to the high sensitivity but limited specificity of the models. While this is a reasonable conclusion, there are still concerns that the high false-positive rate means that each examination will produce several aneurysm candidates requiring review.

Gu *et al* performed a systematic review and meta-analysis of 19 studies using deep learning models for the detection of cerebral aneurysms.^78^ The authors highlight a lack of high-quality prospective research and acknowledge that because there is a lack of data and description, there are many risks of bias and concerns for applicability. While we agree with this, we consider their quality assessment underestimates these studies’ bias and applicability concerns. However, we would caution one of their conclusions that deep learning models can improve clinicians’ reading time, based on the analysis of less than a third of their included studies that measure reading time. Furthermore, in studies showing an improved reading time with deep learning models, it has been highlighted by others that caution should be applied.^71^ For example, it has been highlighted that first, the reading time is of neither clinical nor statistical significance (3.6 s) and second, that there are concerns regarding study methodology as it is unclear how a reader could review each case with 13.8 false-positives per case in 30 s given the number of source images and reformats required (to achieve a sensitivity of 97.5%).^71^

Both systematic reviews also do not encompass machine learning models other than DL. We have found that other machine learning models appear to deliver comparable performance and include one used in a prospective study of 2701 cases performed over 39 months.^55^ The performance accuracy of DL models in particular benefits from large datasets; therefore, one reason for the similarity in performance with other machine learning models may be due to insufficiently sized training datasets. It is noteworthy that the diagnostic accuracy and quality of studies using DL do not appear to be superior to other machine learning models in recent systematic reviews of other AI biomarkers with small datasets.^66 79^

Another review highlighted studies using AI models for rupture risk stratification and outcome prediction,^80^ which is beyond the scope of the current analysis. Our findings confirm that for aneurysm detection using AI CAD, conclusions are limited due to study bias, and that AI CAD performance is compromised by high false-positive rates.

### Implications for future research and clinical practice

AI CAD tools for aneurysm detection are not ready for incorporation into routine clinical practice due to the low level of evidence supporting their use.^67^ Those AI CAD tools that have been evaluated with internal test sets would contribute more to the evidence base if they are re-evaluated in further studies using prospective external data.^81^ Further studies where AI CAD tools are trained on a large and representative dataset and evaluated on a prospective multicenter cohort are needed to clinically validate the efficacy of these tools.^64^

## Conclusion

A range of AI CAD tools designed to automatically detect cerebral aneurysms have been developed and demonstrate promising diagnostic accuracy. However, despite advancements in AI methodology, limited conclusions can be made from the current evidence due to an ongoing high risk of bias and concerns regarding applicability. To ensure clinical adoption, large and representative datasets should be used in studies developing AI CAD tools, with subsequent clinical validation achieved through prospective multicenter studies.

10.1136/jnis-2022-019456.supp3 Supplementary data

## Data Availability

Data are available upon reasonable request. Data analyzed during the study are available from the corresponding author by request.
