# Supplementary material for: Detection of cerebral aneurysms using artificial intelligence: a systematic review and meta-analysis
Source: J Neurointerv Surg. 2022 Nov 14;15(3):262–71. doi: 10.1136/jnis-2022-019456 (PMC9985742; doi:10.1136/jnis-2022-019456)
Supplement: Supplementary data [file jnis-2022-019456supp001.pdf]

Supplemental Material

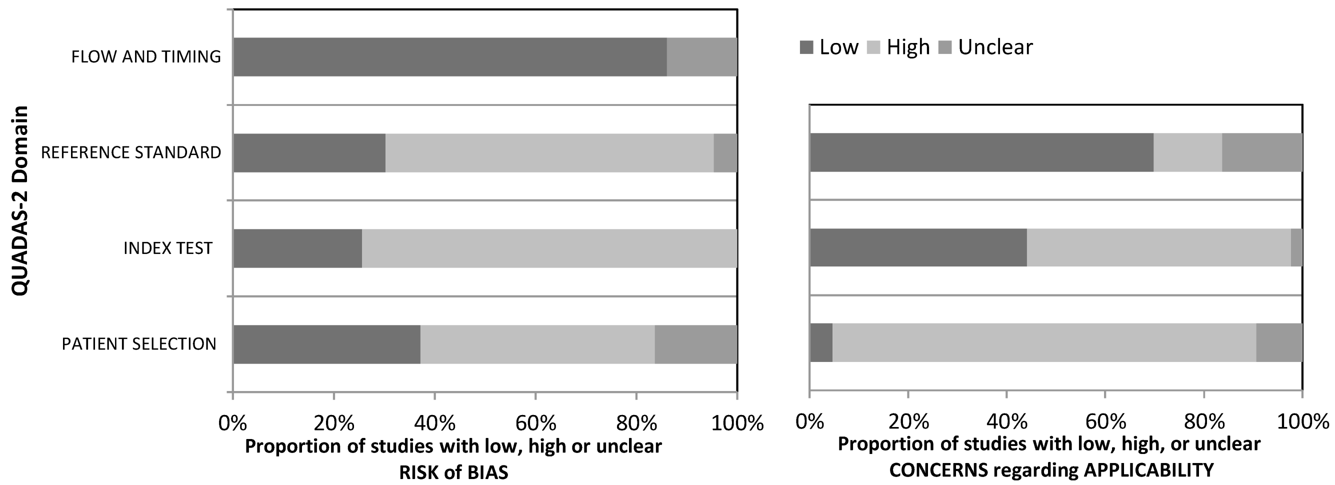

Supplemental figure 1: Summary of quality assessment of studies using the Quality Assessment of Diagnostic Accuracy Studies Two (QUADAS 2) tool.

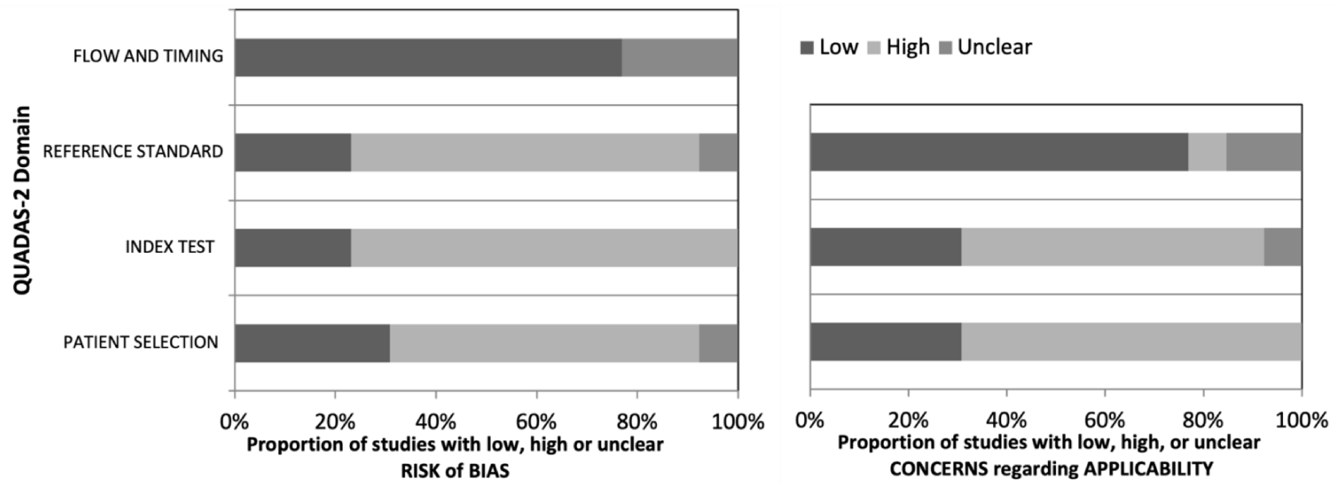

Supplemental figure 2: Summary of quality assessment of studies using the Quality Assessment of Diagnostic Accuracy Studies Two (QUADAS 2) tool for studies published before 2018.

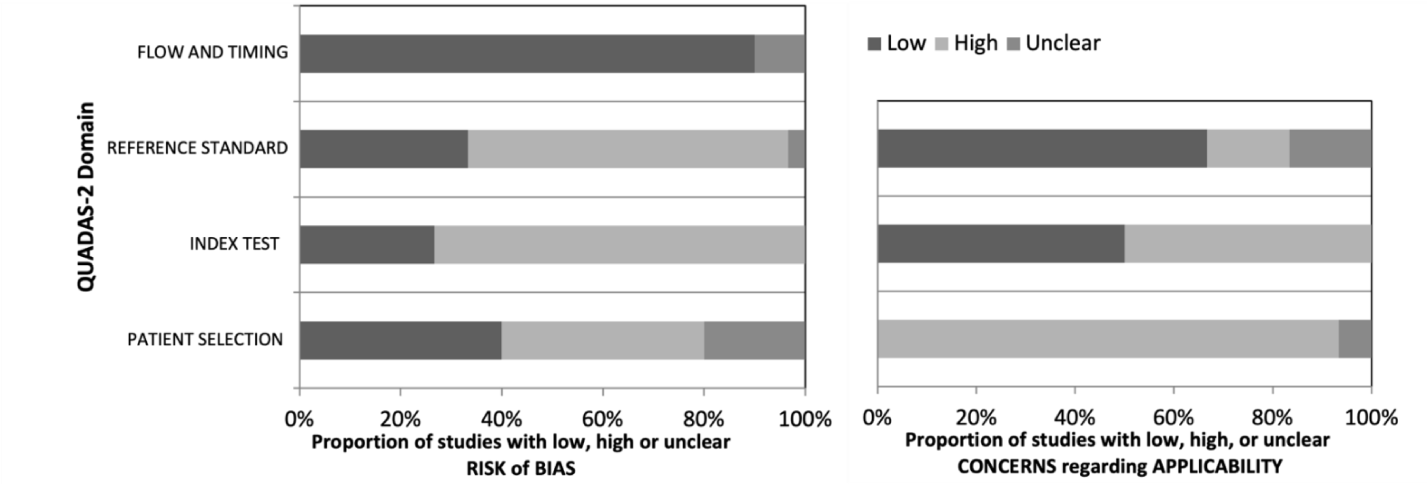

Supplemental figure 3: Summary of quality assessment of studies using the Quality Assessment of Diagnostic Accuracy Studies Two (QUADAS 2) tool for studies published in 2018 or later.

Supplemental Table 1. MEDLINE, EMBASE, Web of Science and Cochrane Register search strategies.

MEDLINE (OVID)<br>1. exp magnetic resonance imaging/<br>2. (mri or mr or (magnetic adj1 resonance)).ab,ti.<br>3. exp Tomography, X-Ray Computed/<br>4. (ct or (comput* adj1 tomograph*)).ab,ti.<br>5. (cat adj3 (scan* or imag* or stud*)).ab,ti.<br>6. (cta or mdcta or (comput* adj1 tomograph* adj3 angiogra*) or (ct adj1 angiogra*) or ((c-arm or 3d or rotational) adj3 angiogra*)).ab,ti.<br>7. (mra or (magnetic adj1 resonance adj1 angiogra*) or (mr adj1 angiogra*) or (mra adj1 (tof or "time of flight")) or ((tof or "time of flight") adj3 angiogra*)).ab,ti.<br>8. (angiogra* or dsa or 3dsa).ab,ti.<br>9. neuroimag*.ti,ab.<br>10. 1 or 2 or 3 or 4 or 5 or 6 or 7 or 8 or 9<br>11. (brain or head or skull or $cerebral or $cerebrum or $cranial or $cranium).ab,ti.<br>12. (detect* or classif* or identif* or diagnos* or predict* or decision or decis* or decid*).ti,ab.<br>13. aneurysm*.ti,ab.<br>14. 10 and 11 and 12 and 13<br>15. exp Diagnosis, Computer-Assisted/ or exp Algorithms/ or exp Artificial Intelligence/ or exp Machine Learning/ or exp Neural Networks, Computer/ or exp Pattern Recognition, Automated<br>16. ((artificial adj1 intelligence) or ((deep or machine) adj1 learning)).mp.<br>17. algorithm*.ab,ti,kw,kf. or automat*.ab,ti. or radiomic*.mp. or (comput* adj3 (aid* or assist* or vision*)).ti,ab.<br>18. ((supervised or unsupervised or (semi adj1 supervised) or deep hybrid or cluster* or bayes* or gauss*) adj3 (learning or model* or net* or algo*)).mp.<br>19. ((feature adj3 (engineer* or select* or extract* or learn*)) or hyperparameter).mp.<br>20. (((neural or conv*) adj1 (net* or learn* or model*)) or CNN or convnet or RNN, or long short-term memory or lstm or gate* recurrent unit or gru or boltzmann machine or deep belief net* or spatial transformer net* or sum product network).mp.<br>21. ((ensemble or transfer or zero shot or reinforcement or dictionary) adj1 (learning or model* or net* or algo*)).mp.<br>22. (vector machine or SVM or ((classification or regression or probability or decision) adj1 tree*) or random forest).mp.<br>23. (generative model* or autoencod* or aae or vae or cae or dae or sdae or gan or generative adversarial).mp.<br>24. (pca or principal component analysis or (k adj1 means) or (nearest adj1 neighbo?r) or knn or (fuzzy adj3 logi*) or isolation forest or hidden markov model or association rule* or feature bag* or score normali$ation).mp.<br>25. 15 or 16 or 17 or 18 or 19 or 20 or 21 or 22 or 23 or 24<br>22. 14 and 25
EMBASE (OVID)<br>1. exp nuclear magnetic resonance imaging/<br>2. (mri or mr or (magnetic adj1 resonance)).ab,ti.<br>3. exp Tomography, X-Ray Computed/<br>4. (ct or (comput* adj1 tomograph*)).ab,ti.

5. (cat adj3 (scan* or imag* or stud*)).ab,ti.
6. (cta or mdcta or (comput* adj1 tomograph* adj3 angiogra*) or (ct adj1 angiogra*) or ((c-arm or 3d or rotational) adj3 angiogra*)).ab,ti.
7. (mra or (magnetic adj1 resonance adj1 angiogra*) or (mr adj1 angiogra*) or (mra adj1 (tof or "time of flight")) or ((tof or "time of flight") adj3 angiogra*)).ab,ti.
8. (angiogra* or dsa or 3dsa).ab,ti.
9. neuroimag*.ti,ab.
10. 1 or 2 or 3 or 4 or 5 or 6 or 7 or 8 or 9
11. (brain or head or skull or $cerebral or $cerebrum or $cranial or $cranium).ab,ti.
12. (detect* or classif* or identif* or diagnos* or predict* or decision or decis* or decid*).ti,ab.
13. aneurysm*.ti,ab.
14. 10 and 11 and 12 and 13
15. computer assisted diagnosis/ or exp algorithm/ or exp artificial intelligence/ or exp machine learning/
16. ((artificial adj1 intelligence) or ((deep or machine) adj1 learning)).mp.
17. (algorithm* or automat*).ab,ti. or radiomic*.mp. or (comput* adj3 (aid* or assist* or vision*)).ti,ab.
18. ((supervised or unsupervised or (semi adj1 supervised) or deep hybrid or cluster* or bayes* or gauss*) adj3 (learning or model* or net* or algo*)).mp.
19. ((feature adj3 (engineer* or select* or extract* or learn*)) or hyperparameter).mp.
20. (((neural or conv*) adj1 (net* or learn* or model*)) or CNN or convnet or RNN, or long short-term memory or lstm or gate* recurrent unit or gru or boltzmann machine or deep belief net* or spatial transformer net* or sum product network).mp.
21. ((ensemble or transfer or zero shot or reinforcement or dictionary) adj1 (learning or model* or net* or algo*)).mp.
22. (vector machine or SVM or ((classification or regression or probability or decision) adj1 tree*) or random forest).mp.
23. (generative model* or autoencod* or aae or vae or cae or dae or sdae or gan or generative adversarial).mp.
24. (pca or principal component analysis or (k adj1 means) or (nearest adj1 neighbo?r) or knn or (fuzzy adj3 logi*) or isolation forest or hidden markov model or association rule* or feature bag* or score normali$ation).mp.
25. 15 or 16 or 17 or 18 or 19 or 20 or 21 or 22 or 23 or 24
22. 14 and 25

Web of Science

1. TI =(mri or mr or (magnetic NEAR/0 resonance)) OR AB=(mri or mr or (magnetic NEAR/0 resonance))
2. TI=(ct or (comput* NEAR/0 tomograph*)) OR AB=(ct or (comput* NEAR/0 tomograph*))
3. TI=(cat NEAR/2 (scan* or imag* or stud*)) OR AB=(cat NEAR/2 (scan* or imag* or stud*))
4. TI=(neuroimag*) OR AB=(neuroimag*)
5. TI = (cta or mdcta or (comput* adj1 tomograph* adj3 angiogra*) or (ct adj1 angiogra*) or ((c-arm or 3d or rotational) adj3 angiogra*)) or AB = (cta or mdcta or (comput* adj1 tomograph* adj3 angiogra*) or (ct adj1 angiogra*) or ((c-arm or 3d or rotational) adj3 angiogra*))
6. TI = (mra or (magnetic adh1 resonance adh1 angiogra*) or (mr adh1 angiogra*) or (mra adh1 (tof or "time of flight")) or ((tof or "time of flight") adh3 angiogra*)) OR AB = (mra or (magnetic adh1 resonance adh1 angiogra*) or (mr adh1 angiogra*) or (mra adh1 (tof or "time of flight")) or ((tof or "time of flight") adh3 angiogra*))

7. TI=(angiogra* or DSA or 3DSA) OR AB=(angiogra* or DSA or 3DSA)
8. #1 OR #2 OR #3 OR #4 OR #5 OR #6 OR #7
9. **TI = (brain or head or skull or cerebral or intracerebral or cerebrum or cranial or intracranial or cranium) OR AB = (brain or head or skull or cerebral or intracerebral or cerebrum or cranial or intracranial or cranium)**
10. TI=(detect* or classif* or identif* or diagnos* or predict* or decision or decid*) OR AB=(detect* or classif* or identif* or diagnos* or predict* or decision or decid*)
11. TI=(aneurysm*) OR AB=(aneurysm*)
12. #8 AND #9 AND #10 AND #11
13. TI=((artificial NEAR/0 intelligence) or ((deep or machine) NEAR/0 learning)) OR AB=((artificial NEAR/0 intelligence) or ((deep or machine) NEAR/0 learning))
14. TI=(algorithm* or automat* or radiomic* or (comput* NEAR/2 (aid* or assist* or vision*))) OR AB=(algorithm* or automat* or radiomic* or (comput* NEAR/2 (aid* or assist* or vision*)))
15. TI = (supervised NEAR/2 (learning or model* or net* or algo*)) OR AB = (supervised NEAR/2 (learning or model* or net* or algo*))
16. TI = (unsupervised NEAR/2 (learning or model* or net* or algo*)) OR AB= (unsupervised NEAR/2 (learning or model* or net* or algo*))
17. TI=(semi supervised NEAR/2 (learning or model* or net* or algo*)) OR AB=(semi supervised NEAR/2 (learning or model* or net* or algo*))
18. TI=(deep hybrid NEAR/2 (learning or model* or net* or algo*)) OR AB=(deep hybrid NEAR/2 (learning or model* or net* or algo*))
19. TI=(bayes* NEAR/2 (learning or model* or net* or algo*)) OR AB=(bayes* NEAR/2 (learning or model* or net* or algo*))
20. TI=(cluster* NEAR/2 (learning or model* or net* or algo*)) OR AB=(cluster* NEAR/2 (learning or model* or net* or algo*))
21. TI=(gauss* NEAR/2 (learning or model* or net* or algo*)) OR AB=(gauss* NEAR/2 (learning or model* or net* or algo*))
22. TI=((feature NEAR/2 (engineer* or select* or extract* or learn*)) or hyperparameter) OR AB=((feature NEAR/2 (engineer* or select* or extract* or learn*)) or hyperparameter)
23. TI=(((neural or conv*) NEAR/0 (net* or learn* or model*)) or CNN or convnet or RNN, or long short-term memory or lstm or gate* recurrent unit or gru or boltzmann machine or deep belief net* or spatial transformer net* or sum product network) OR AB=(((neural or conv*) NEAR/0 (net* or learn* or model*)) or CNN or convnet or RNN, or long short-term memory or lstm or gate* recurrent unit or gru or boltzmann machine or deep belief net* or spatial transformer net* or sum product network)
24. TI=(ensemble NEAR/0 (learning or model* or net* or algo*)) OR AB=(ensemble NEAR/0 (learning or model* or net* or algo*))
25. TI=(transfer NEAR/0 (learning or model* or net* or algo*)) OR AB=(transfer NEAR/0 (learning or model* or net* or algo*))
26. TI=(zero shot NEAR/0 (learning or model* or net* or algo*)) OR AB=(zero shot NEAR/0 (learning or model* or net* or algo*))
27. TI=(reinforcement NEAR/0 (learning or model* or net* or algo*)) OR AB=(reinforcement NEAR/0 (learning or model* or net* or algo*))
28. TI=(dictionary NEAR/0 (learning or model* or net* or algo*)) OR AB=(dictionary NEAR/0 (learning or model* or net* or algo*))
29. TI=((vector machine or SVM or ((classification or regression or probability or decision) NEAR/0 tree*) or random forest)) OR AB=((logistic regression or vector machine or SVM or ((classification or regression or probability or decision) NEAR/0 tree*) or random forest))
30. TI=((generative model* or autoencod* or aae or vae or cae or dae or sdae or gan or generative adversarial)) OR AB=((generative model* or autoencod* or aae or vae or cae or dae or sdae or gan or generative adversarial))

31. TI=(pca or principal component analysis or (k near/0 means) or (nearest near/0 neighbo$r) or knn or (fuzzy near/0 logi*) or isolation forest or hidden markov model or association rule* or feature bag* or score normali$ation) OR AB=(pca or principal component analysis or (k near/0 means) or (nearest near/0 neighbo$r) or knn or (fuzzy near/0 logi*) or isolation forest or hidden markov model or association rule* or feature bag* or score normali$ation)
32. #31 OR #30 OR #29 OR #28 OR #27 OR #26 OR #25 OR #24 OR #23 OR #22 OR #21 OR #20 OR #19 OR #18 OR #17 OR #16 OR #15 OR #14 OR #13
33. #32 AND #12

Cochrane Register

ID	Search
#1	MeSH descriptor: [Magnetic Resonance Imaging] explode all trees
#2	(mri or mr or (magnetic adj1 resonance)):ab,ti
#3	MeSH descriptor: [Tomography, X-Ray Computed] explode all trees
#4	(ct or (comput* adj1 tomograph*)):ab,ti
#5	(cat adj3 (scan* or imag* or stud*)):ab,ti
#6	(cta or mdcta or (comput* adj1 tomograph* adj3 angiogra*) or (ct adj1 angiogra*) or ((c-arm or 3d or rotational) adj3 angiogra*)):ab,ti
#7	(mra or (magnetic adj1 resonance adj1 angiogra*) or (mr adj1 angiogra*) or (mra adj1 (tof or "time of flight")) or ((tof or "time of flight") adj3 angiogra*)):ab,ti
#8	(angiogra* or DSA or 3DSA):ab,ti
#9	neuroimag*:ti,ab
#10	(3-#9)
#11	(**brain or head or skull or cerebral or intracerebral or cerebrum or cranial or intracranial or cranium):** ti,ab
#12	aneurysm*:ti,ab
#13	(detect* or classif* or identif* or diagnos* or predict* or decision or decis* or decid*):ti,ab
#14	(10-#13)
#15	MeSH descriptor: [Diagnosis, Computer-Assisted] explode all trees
#16	MeSH descriptor: [Algorithms] explode all trees
#17	MeSH descriptor: [Artificial Intelligence] explode all trees
#18	MeSH descriptor: [Neural Networks, Computer] explode all trees
#19	MeSH descriptor: [Machine Learning] explode all trees
#20	((artificial adj1 intelligence) or ((deep or machine) adj1 learning))
#21	algorithm*:ab,ti,kw or automat*:ab,ti or radiomic*:ab,ti,kw or (comput* adj3 (aid* or assist* or vision*)):ab,ti
#22	((supervised or unsupervised or (semi adj1 supervised) or deep hybrid or cluster* or bayes* or gauss*) adj3 (learning or model* or net* or algo*))
#23	((feature adj3 (engineer* or select* or extract* or learn*)) or hyperparameter)
#24	(((neural or conv*) adj1 (net* or learn* or model*)) or CNN or convnet or RNN, or long short-term memory or lstm or gate* recurrent unit or gru or boltzmann machine or deep belief net* or spatial transformer net* or sum product network)
#25	((ensemble or transfer or zero shot or reinforcement or dictionary) adj1 (learning or model* or net* or algo*))
#26	(vector machine or SVM or ((classification or regression or probability or decision) adj1 tree*) or random forest)
#27	(generative model* or autoencod* or aae or vae or cae or dae or sdae or gan or generative adversarial)

#28	(pca or principal component analysis or (k adj1 means) or (nearest adj1 neighbo?r) or knn or (fuzzy adj3 logi*) or isolation forest or hidden markov model or association rule* or feature bag* or score normali$ation)
#29	(11-#28)
#30	#14 AND #29

Supplemental statistical information

The bivariate random-effect model allows for two key scenarios[1-4]. First, the principal diagnostic accuracy measures tend to be related to one another through the cut-off value. The sensitivity is usually negatively correlated to the specificity. Second, a relatively high degree of heterogeneity is seen among the results of diagnostic studies. This can be addressed using the bivariate model which assumes that the logits of the diagnostic accuracy quantities follow a bivariate normal distribution, allowing for a non-zero correlation.

The relevant computations behind both the bivariate random-effects model and the univariate analysis are conducted on the data transformed to logit units[5]. Initially, a logit scale is used for the pooled estimates, but this is then converted back to the original probability scale (0-1) to aid interpretability.

References

1. Booth TC, Grzeda M, Chelliah A, et al. Imaging Biomarkers of Glioblastoma Treatment Response: A Systematic Review and Meta-Analysis of Recent Machine Learning Studies. *Front Oncol* 2022;12:799662. doi: 10.3389/fonc.2022.799662 [published Online First: 20220131]
2. Zhou X-H, Obuchowski NA, McClish DK. Statistical methods in diagnostic medicine. Second ed. Chichester: John Wiley & Sons 2011.
3. Harbord R, Whiting P. Metandi: Meta-analysis of Diagnostic Accuracy Using Hierarchical Logistic Regression. *Stata Journal* 2009;9:211-29. doi: 10.1177/1536867X0900900203
4. Schwarzer G, Carpenter JR, Rücker G. Meta-analysis with R: Springer 2015.
5. Wang N. How to Conduct a Meta-Analysis of Proportions in R: A Comprehensive Tutorial2018.
