# Supplementary material for: Detection of cerebral aneurysms using artificial intelligence: a systematic review and meta-analysis
Source: J Neurointerv Surg. 2022 Nov 14;15(3):262–71. doi: 10.1136/jnis-2022-019456 (PMC9985742; doi:10.1136/jnis-2022-019456)
Supplement: Supplementary data [file jnis-2022-019456supp002.pdf]

Supplemental Figures

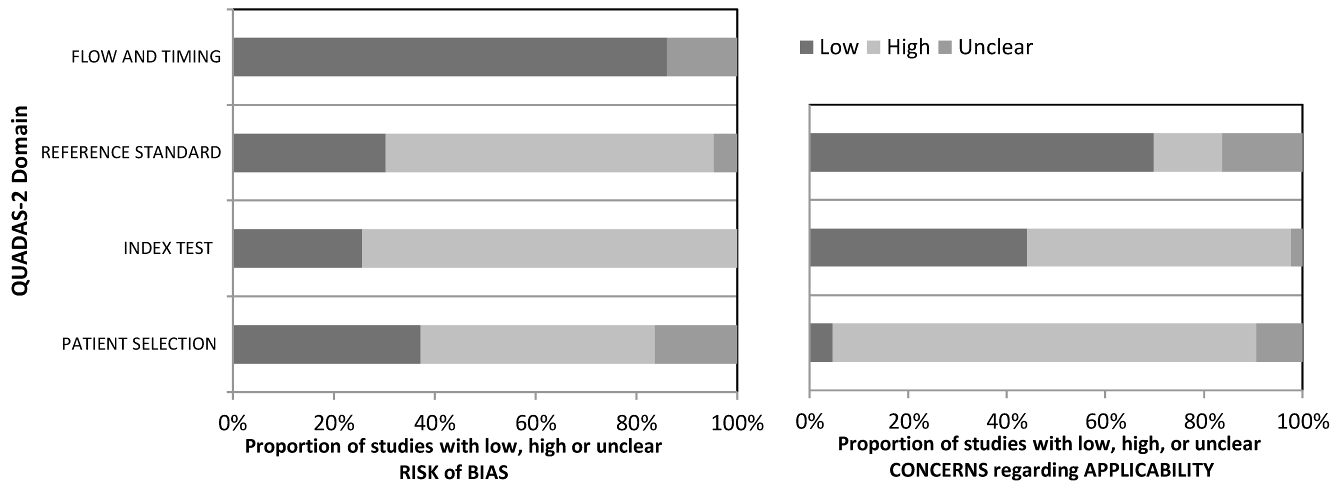

Supplemental figure 1: Summary of quality assessment of studies using the Quality Assessment of Diagnostic Accuracy Studies Two (QUADAS 2) tool.

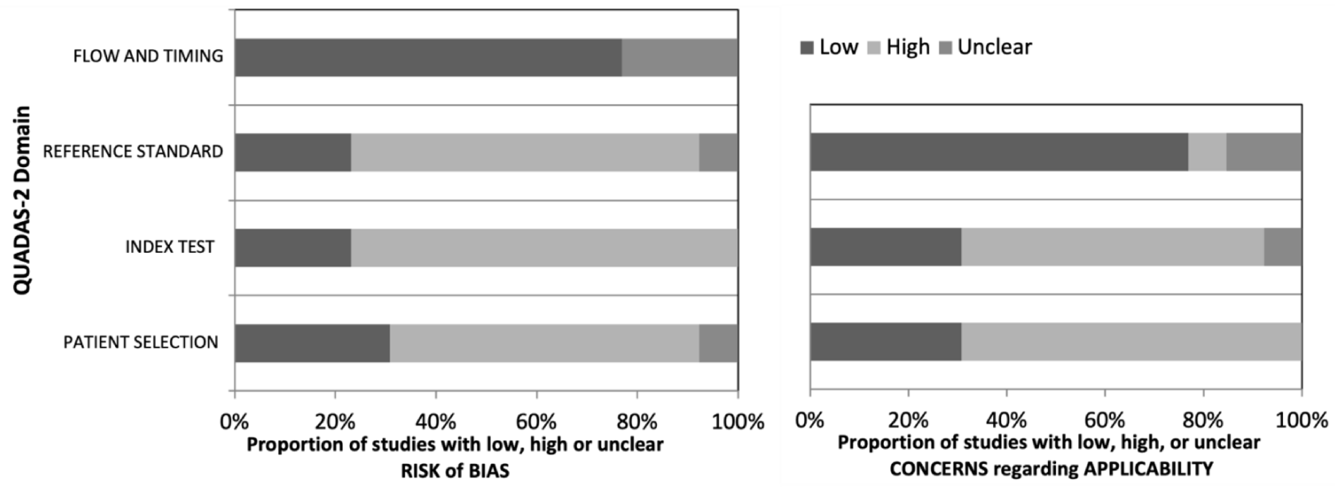

Supplemental figure 2: Summary of quality assessment of studies using the Quality Assessment of Diagnostic Accuracy Studies Two (QUADAS 2) tool for studies published before 2018.

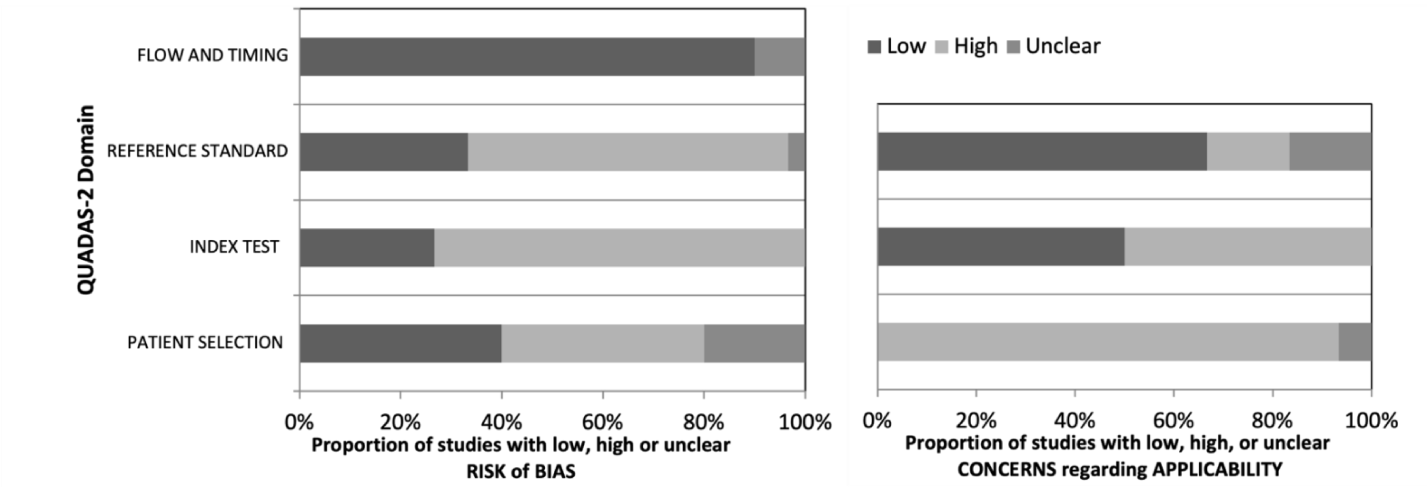

Supplemental figure 3: Summary of quality assessment of studies using the Quality Assessment of Diagnostic Accuracy Studies Two (QUADAS 2) tool for studies published in 2018 or later.
